# Integrated bioinformatics analysis of molecular signatures and therapeutic targets in early sepsis

**DOI:** 10.3389/fcimb.2026.1802215

**Published:** 2026-03-23

**Authors:** Xiaowei Gai, Yaqing Li, Yanan Wang, Dan Gao, Shanshan Wu, Yanan Geng, Jiamin Zhang, Minghui Yao, Gaiqi Yao, Qiuyan Wang

**Affiliations:** 1 Department of Critical Care Medicine, Qinhuangdao Hospital of Peking University Third Hospital, Qinhuangdao, Hebei, China; 2 Department of Infectious Diseases, Hebei General Hospital, Shijiazhuang, Hebei, China; 3 Department of Critical Care Medicine, Peking University Third Hospital, Beijing, China

**Keywords:** bioinformatics, biomarkers, drug repurposing, immunology, sepsis, transcriptomics

## Abstract

**Objective:**

This study aimed to identify key molecular signatures and therapeutic targets in early sepsis through integrated bioinformatics analysis.

**Methods:**

We analyzed three independent blood transcriptomic datasets (GSE95233, GSE137340, GSE57065) from the Gene Expression Omnibus. Differential expression, functional enrichment, and protein-protein interaction network analyses were performed to identify overlapping differentially expressed genes (DEGs) and hub genes. The Connectivity Map (cMAP) database was queried to predict potential therapeutic compounds. An independent dataset validated the diagnostic performance and cellular correlates of the hub genes.

**Results:**

We identified 330 overlapping differentially expressed genes enriched in immune pathways like T-cell receptor signaling. Ten hub genes central to T-cell function were pinpointed (CD4, CD247, CD3E, CD2, FYN, ZAP70, CD3G, ITK, LAT, CD5). Independent validation confirmed that these hub genes were significantly down-regulated in sepsis patients, with their expression levels strongly correlating with T-cell exhaustion and inversely correlating with myeloid cell infiltration. While these genes demonstrated excellent diagnostic accuracy (AUC: 0.908–0.999), this high predictive performance likely reflects differences in immune cell composition rather than disease-specific molecular signatures. Additionally, cMAP analysis predicted six potential therapeutic agents (e.g., anastrozole, etofenamate), offering a theoretical framework for drug repurposing in sepsis.

**Conclusion:**

This study independently validates the significant down-regulation of T-cell-related genes in early sepsis, reflecting a profound disruption of adaptive immunity. Our findings confirm the robustness of these genes as surrogate markers of early immune imbalance, establishing a critical foundation for future investigations into T-cell-focused immunomodulatory strategies in sepsis.

## Introduction

Sepsis and septic shock represent critical medical emergencies defined by an aberrant host response to infection, resulting in potentially fatal organ dysfunction (Lin et al., 2024). Moreover, these conditions pose a substantial global health issue, marked by elevated mortality rates and significant long-term health complications among survivors (Muhammad et al., 2024). The financial implications for healthcare systems are severe. These are further exacerbated by the challenges associated with timely diagnosis and the currently limited treatment options accessible to healthcare providers (Brar et al., 2025). Conventional diagnostic approaches predominantly depend on clinical criteria and non-specific biomarkers, which frequently lack the precision required for effective intervention. Consequently, there is an urgent demand for innovative early biomarkers and therapeutic targets in the management of sepsis, underscoring the necessity for further investigation in this domain (Reisinger et al., 2021).

To bridge this gap, recent research initiatives in sepsis have increasingly aimed to elucidate the fundamental molecular mechanisms, particularly through transcriptomic analyses using RNA sequencing (RNA-seq) technology (Cheng et al., 2021). These investigations have revealed a multitude of differentially expressed genes (DEGs) linked to immune dysregulation in sepsis, especially involving T cells and macrophages, emphasizing the critical interplay between hyperinflammation and immunosuppression in its pathophysiology (Yao et al., 2022; Rahmel et al., 2024). Nevertheless, despite the identification of numerous DEGs, the establishment of robust and reproducible gene signatures for diagnostic and therapeutic applications remains challenging, largely due to inter-study heterogeneity and inconsistent findings across studies. In particular, the correlation between specific gene expression profiles - especially those related to T-cell functionality - and the severity of sepsis or patient outcomes has not been adequately substantiated (Kim et al., 2021; Tian et al., 2023). These limitations underscore a critical gap in the current literature: the lack of validated overlapping DEGs across independent datasets that could serve as reliable biomarkers and therapeutic targets.

The primary aims of this study are to identify essential overlapping DEGs relevant to sepsis, clarify their biological roles, forecast potential therapeutic agents, and validate their diagnostic relevance, as well as their association with immune cell infiltration during the early phases of sepsis. Through these objectives, by identifying key genes and potential therapies, we aim to deepen the understanding of the pathophysiology of sepsis and provide valuable insights that may lead to improved clinical outcomes for patients afflicted by this grievous condition. By employing a range of bioinformatics methodologies, we seek to lay the groundwork for the creation of innovative diagnostic and therapeutic strategies aimed at optimizing the management of sepsis and its related complications.

## Materials and methods

### Data acquisition

In this study, three datasets related to sepsis were obtained from the Gene Expression Omnibus (GEO) database (https://www.ncbi.nlm.nih.gov/geo) (Alameer and Chicco, 2022). The selection process adhered to specific criteria: (1) a confirmed diagnosis of sepsis or septic shock; (2) participants aged 18 years or older; (3) the presence of clinical and expression data collected within the first 24 hours following the onset of the condition. The datasets incorporated in this research were GSE95233, GSE137340, and GSE57065, all of which fulfilled these prerequisites. The GSE95233 dataset (platform: GPL570) consists of whole blood samples from 51 individuals diagnosed with septic shock and 22 healthy controls. The GSE137340 dataset (platform: GPL10558) includes whole blood samples from 27 sepsis patients alongside 12 healthy donors. To ensure the robustness and reproducibility of our findings, GSE95233 and GSE137340 were designated as the training datasets, while GSE57065, which comprises 28 septic shock patients and 25 healthy controls, was utilized for validation purposes.

### Data preprocessing

The Series Matrix files underwent processing and normalization through the robust multiarray average (RMA) method (Sahly et al., 2021) implemented in R (version 4.5.2). To correct for batch effects across the different GSE datasets, the ComBat function from the ‘sva’ R package (version 3.58.0) was subsequently applied to the normalized expression data, ensuring comparability and mitigating non-biological technical variations prior to downstream analysis. Following this, the probe expression matrix was log2-transformed. Probe IDs were aligned with gene symbols utilizing the relevant Bioconductor annotation package, and the expression values for duplicate genes were averaged (Hussain et al., 2025). Differentially expressed genes (DEGs) were identified using the limma package(version 3.66.0) in R, applying significance thresholds of |logFC| > 1 and an adjusted *P*-value < 0.05. To visualize the DEGs, volcano plots were created using the ggplot2 package in R. Common DEGs that were either up-regulated or down-regulated between the GSE95233 and GSE137340 datasets were determined using an online Venn diagram tool (https://bioinfogp.cnb.csic.es/tools/venny/) (Davies and McLaren, 2024). Gene Ontology (GO) serves as a prominent bioinformatics resource for gene annotation and the exploration of biological processes associated with these genes, which encompass molecular function (MF), biological processes (BPs), and cellular composition (CC) (Liu et al., 2023). The Kyoto Encyclopedia of Genes and Genomes (KEGG) functions as a database resource aimed at elucidating complex functions and biological systems by creating extensive molecular datasets through high-throughput experimental methodologies (Kanehisa et al., 2025). To pinpoint target genes, we conducted GO annotation and KEGG pathway enrichment analysis for hub mRNA predictions using the Xiantao academic tool (https://www.xiantao.love/products/). A *p*-value threshold of < 0.05 was employed to signify a statistically significant outcome.

### Establishment of protein–protein interaction networks

Protein–Protein Interaction (PPI) networks serve as mathematical frameworks that illustrate the physical interactions among gene candidates at the protein level, facilitating a deeper understanding of disease mechanisms and aiding in drug discovery efforts (Yang et al., 2020). In this study, PPI networks corresponding to commonly identified differentially expressed genes (DEGs) were constructed utilizing the STRING database (http://string-db.org), applying a threshold for the minimum required interaction score (combined score > 0.7). The interaction data were subsequently downloaded and visualized through the use of Cytoscape software (version 3.10.3). The topological analysis plugin, CytoHubba, was employed to prioritize hub genes based on various centrality metrics.

### Prediction of therapeutic small molecular agents

Candidate small molecules were identified through the Connectivity Map (cMAP) database provided by the Broad Institute (https://clue.io/) (Lu et al., 2020). To categorize the candidate small molecules aimed at treating sepsis, all commonly shared DEGs from the GSE95233 and GSE137340 datasets were uploaded into the cMAP database. Molecules exhibiting high negative connectivity scores may possess potential therapeutic efficacy against the disease. Detailed information regarding these compounds, including their structural representations, was acquired using PubChem (https://www.ncbi.nlm.nih.gov/) and ChemDraw.

### Validation of hub gene expression

To reduce the likelihood of false positives, the identified hub genes underwent further validation within the independent GSE57065 dataset. Differential expression analysis of the ten genes was conducted between the patient and control cohorts utilizing GraphPad Prism version 8.0.1. Additionally, a principal component analysis (PCA) plot was generated employing the R package ggplot2 to illustrate the distinct expression profiles of septic patients in comparison to healthy donors. A *P*-value of less than 0.05 was deemed indicative of a significant difference between the groups.

### Receiver operating characteristic curves of hub genes

To assess the predictive capability of the identified critical genes regarding disease outcomes, the accuracy of the hub genes was evaluated through ROC analysis, and the area under the curve (AUC) values were calculated using an online platform (https://www.xiantao.love/products/). The efficacy classification was as follows: non-efficient (AUC ≦ 0.5); modestly efficient (0.5 < AUC < 0.7); and highly efficient (AUC > 0.7).

### Immune cell infiltration analysis of critical genes

We analyzed the levels of immune cell infiltration across all samples from septic patients and healthy donors. The single-sample Gene Set Enrichment Analysis (ssGSEA) method was employed to quantify the proportions of 22 distinct immune cell types based on gene expression data (Wang et al., 2021). Utilizing the Xiantao academic online analysis tool, we generated comparative plots and correlation heatmaps for each hub gene in relation to immune cell types.

### Statistical analysis

Statistical analyses and graphical visualizations were performed using R software (version 4.5.2) and GraphPad Prism (version 8.0). For comparisons of continuous variables between two groups, the Student’s t-test was applied for data meeting normality and homogeneity of variance assumptions, while the Wilcoxon rank-sum test was used for non-normally distributed data. Correlation analyses were conducted using either Pearson’s correlation coefficient for normally distributed data or Spearman’s rank correlation coefficient for non-normally distributed data. For differential expression analysis, the Benjamini-Hochberg procedure was applied to control the false discovery rate (FDR). All data are presented as mean ± standard deviation (SD), and a p-value < 0.05 was considered statistically significant.

## Results

### Identification of differentially expressed genes in two datasets

**Figure 1:**
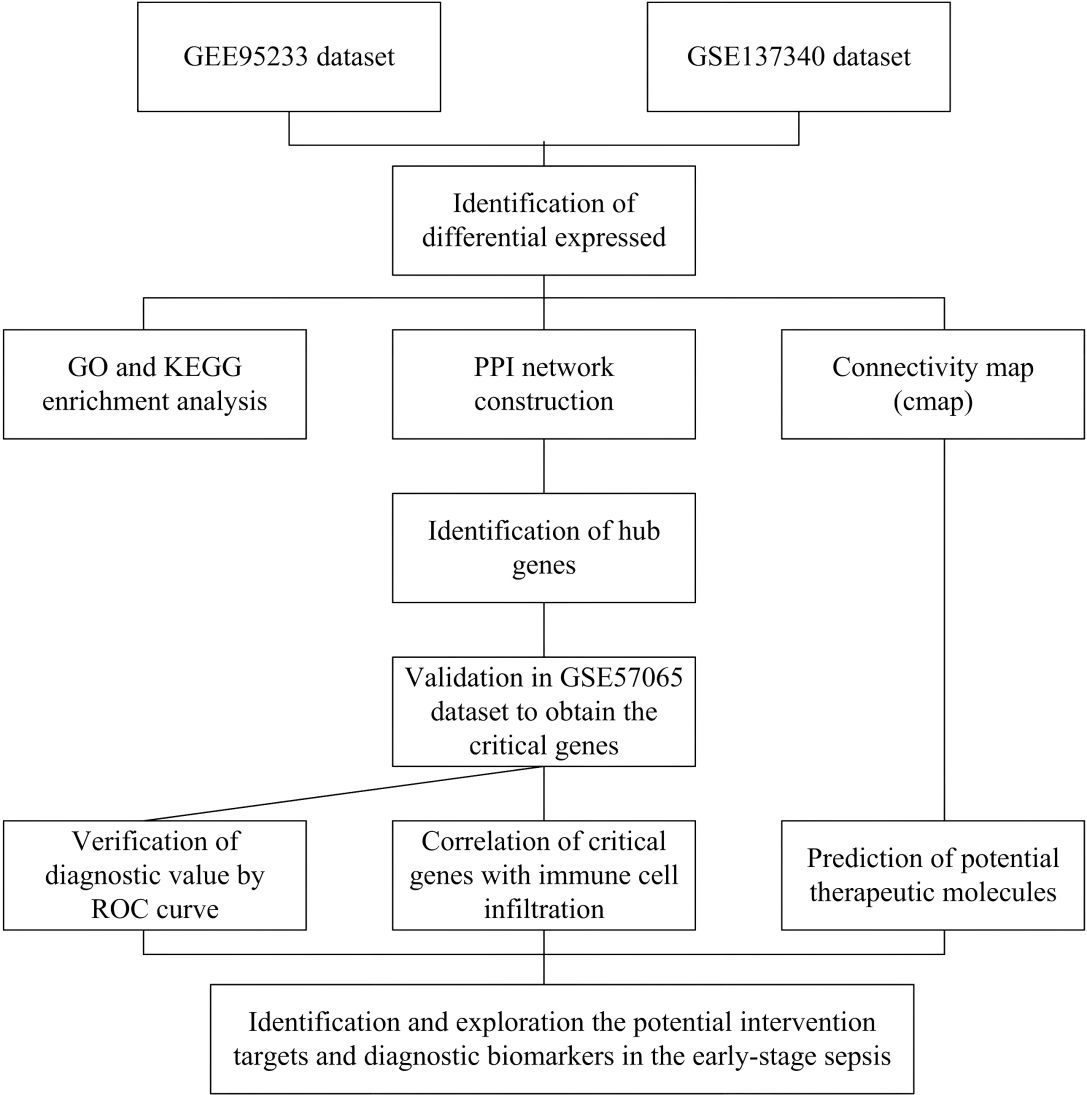
Workflow of this study. PPI, protein–protein interaction; GO, Gene Ontology; KEGG, Kyoto Encyclopedia of Genes and Genomes; ROC, Receiver Operating Characteristic curve.

**Figure 2:**
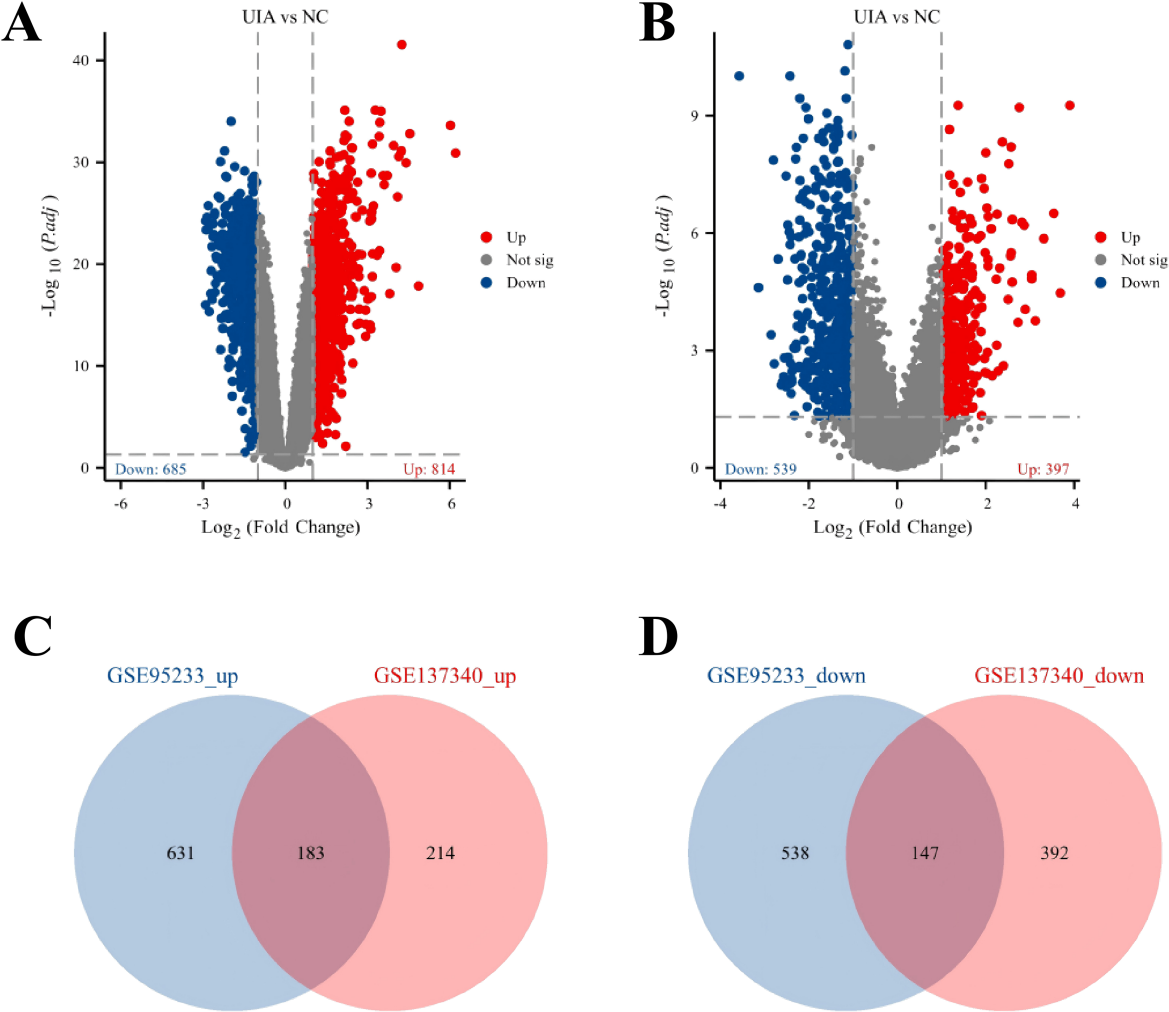
The common genes between GSE95233 and GSE137340. **(A, B)** Volcano plot of all DEGs between sepsis patients on day 1 and healthy controls in **(A)** GSE95233 dataset or **(B)** GSE137340 dataset analyzed by the limma R package, genes with a *P*-value < 0.05 and |logFC| > 1 were considered as significant DEGs, red dots indicate the up-regulated genes, blue dots represent down-regulated genes, and gray dots indicate non-significant genes; **(C, D)** Venn diagram of up- and down-regulated genes in the two datasets.

The methodological framework of the study is illustrated in **Figure 1**. To ascertain the overlapping DEGs, we utilized microarray data from two distinct datasets: GSE95233 and GSE137340, which served as our training sets. Following the normalization and logarithmic transformation of the data, probes lacking annotation information were excluded, and the average expression values were computed in instances of duplicate data using R software. Genes were classified as differentially expressed genes (DEGs) based on the criteria of an adjusted *P*-value < 0.05 and |logFC| > 1. In total, we identified 1499 DEGs within the GSE95233 dataset (comprising 814 upregulated and 685 downregulated genes) and 936 DEGs in the GSE137340 dataset (including 397 upregulated and 539 downregulated genes), which were subsequently visualized in volcano plots as shown in **Figures 2A, B**. Furthermore, we extracted the overlapping DEGs common to both datasets, yielding a total of 183 upregulated and 147 downregulated genes identified as potential crosstalk genes, as represented in the Venn diagrams in **Figures 2C, D**.

### Functional enrichment analysis of the common shared DEGs

**Figure 3:**
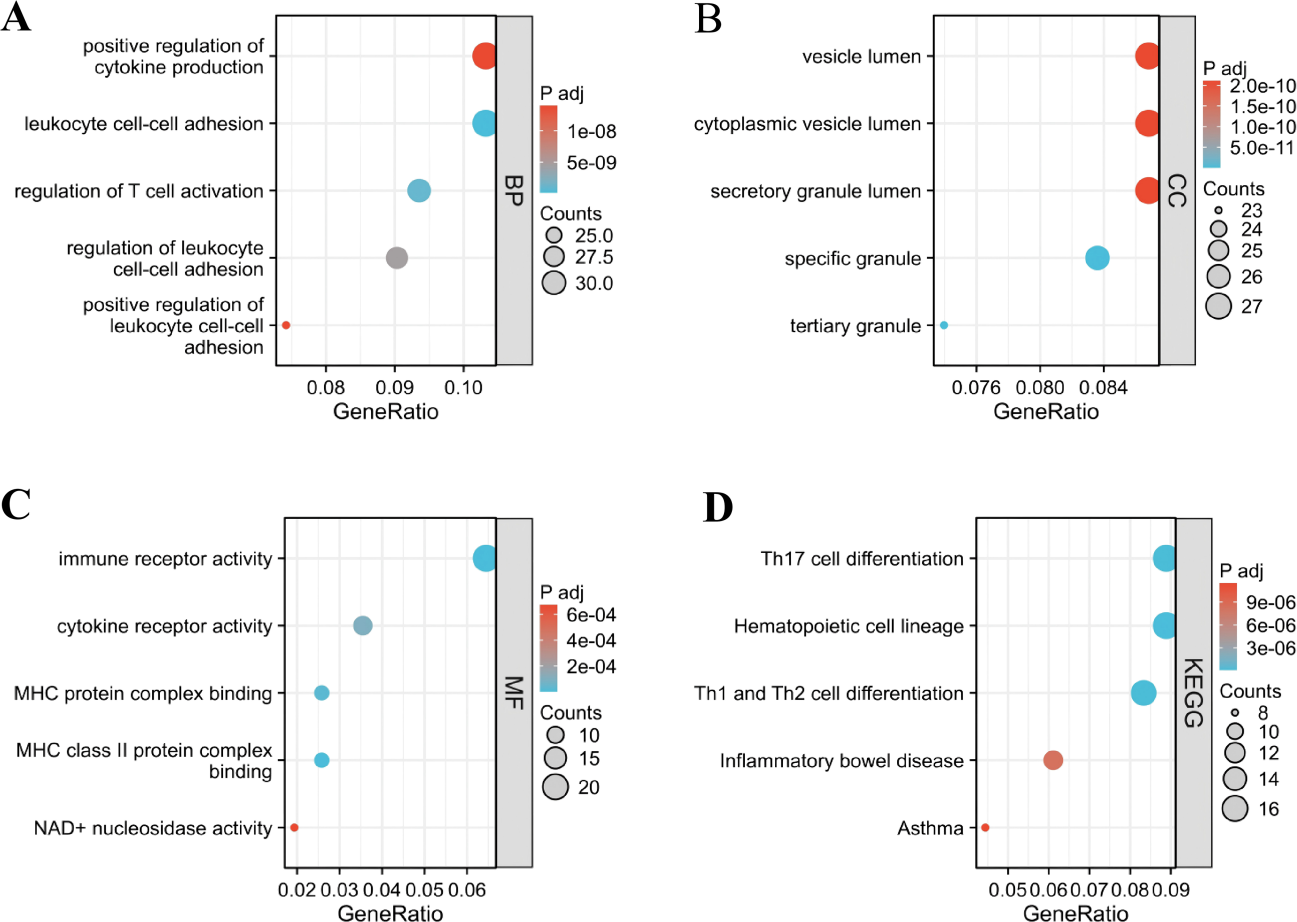
GO anal and KEGG pathways enrichment analysis of the common DEGs. **(A–C)** GO categories of biological process (BP), cellular component (CC), and molecular function (MF), respectively. **(D)** KEGG enrichment analysis of DEGs.

**Figure 3** presents the functional enrichment analysis results for the target genes of pivotal mRNAs. In the Biological Process (BP) category, the identified genes were primarily involved in immune cell activation and cell-cell adhesion, including the regulation of T-cell activation, leukocyte adhesion, cytokine production, and lymphocyte differentiation (**Figure 3A**). Cellular Component (CC) analysis revealed significant enrichment in granule- and vesicle-related structures, such as secretory granules and cytoplasmic vesicle lumens (**Figure 3B**). Molecular Function (MF) analysis indicated that these genes were associated with immune receptor activity, MHC class II protein binding, cytokine receptor activity, NAD+ nucleosidase activity, and magnesium ion binding (**Figure 3C**). KEGG pathway enrichment further demonstrated that the target genes were predominantly involved in immune system-related pathways, including hematopoietic cell lineage, Th1/Th2 and Th17 cell differentiation, inflammatory bowel disease, asthma, and the T-cell receptor signaling pathway (**Figure 3D**).

### Identification of therapeutic small molecular agents based on the DEGs

**Figure 4:**
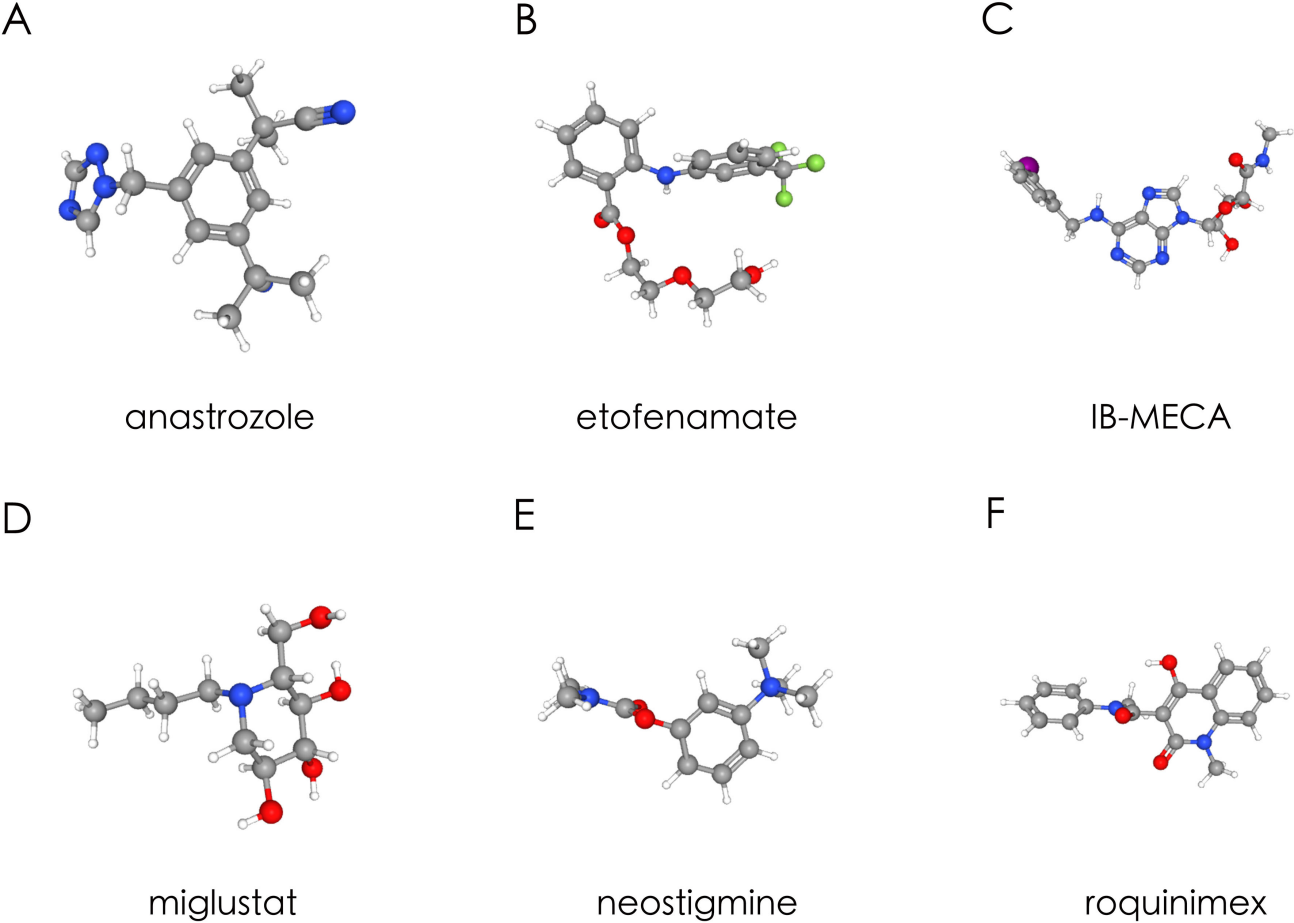
The structure of six small molecules. **(A)** Anastrozole; **(B)** Etofenamate; **(C)** IB-MECA; **(D)** Miglustat; **(E)** Neostigmine and **(F)** Roquinimex.

To explore potential therapeutic avenues suggested by the sepsis-associated transcriptomic signature, we queried the Connectivity Map (cMAP) database to identify small molecules capable of reversing the common differentially expressed gene (DEG) expression pattern observed in sepsis. This analysis, while based on cancer-derived cell line perturbation data and therefore requiring cautious interpretation in the context of systemic inflammatory diseases, yielded six candidate compounds with the highest absolute enrichment values: anastrozole, etofenamate, IB-MECA, miglustat, neostigmine, and roquinimex (**Table 1**). Notably, these agents have been previously associated with general immunomodulatory or anti-inflammatory properties. For instance, anastrozole has demonstrated anti-inflammatory effects in experimental sepsis models, while etofenamate may mitigate systemic inflammation as a non-steroidal anti-inflammatory drug. IB-MECA, an adenosine A3 receptor agonist, could potentially attenuate cytokine storms, and neostigmine may influence cholinergic anti-inflammatory pathways. However, direct mechanistic links between these compounds and the T-cell-related hub genes identified in this study remain to be established. Therefore, these findings should be considered exploratory, providing a theoretical reference for future investigations rather than definitive therapeutic candidates. The chemical structures and three-dimensional conformations of these compounds are presented in **Figure 4** and detailed in PubChem.

**Table 1:** Six small molecules predicted with the common shared DEGs.

Rank	norm_cs	Name	Description
1	-1.9239	IB-MECA	Adenosine A3 receptor agonist
2	-1.9047	Etofenamate	Non-steroidal anti-inflammatory drug (NSAID)
3	-1.8712	Anastrozole	Aromatase inhibitor
4	-1.8599	Neostigmine	Acetylcholinesterase inhibitor
5	-1.8457	Roquinimex	Immunomodulator
6	-1.798	Miglustat	Glucosylceramide synthase inhibitor

### Construction of protein-protein interaction networks and identification of hub genes

**Figure 5:**
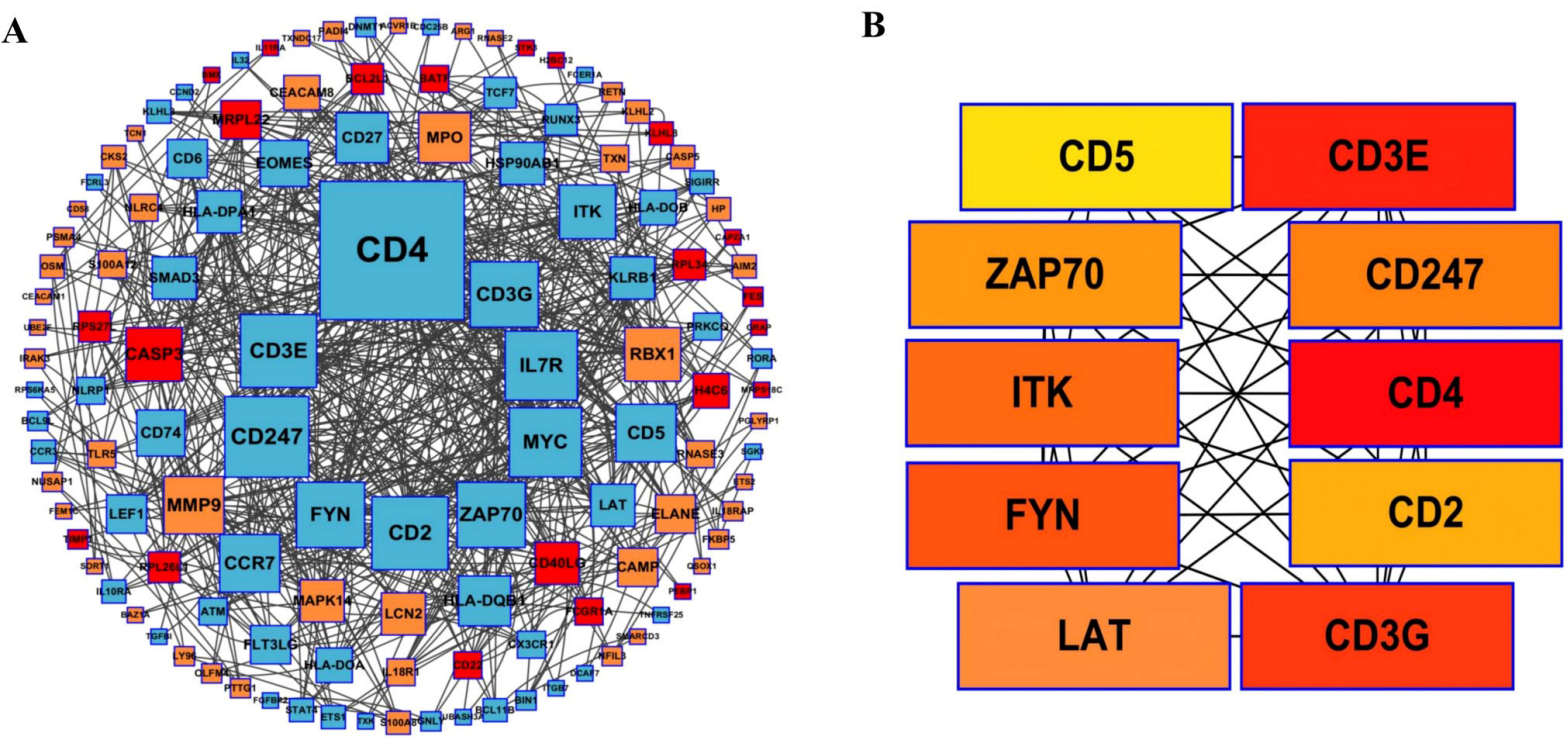
**(A)** The PPI network was constructed and visualized using Cytoscape. The orange boxes indicate the up-regulated genes and the blue boxes represent down-regulated genes. **(B)** The top ten hub genes.

In total, 330 proteins linked to the commonly differentially expressed genes were identified and integrated into the protein-protein interaction network illustrated. To explore their molecular interactions, we constructed a protein-protein interaction (PPI) network using the STRING database, applying a confidence score threshold of greater than 0.7. This approach revealed a sophisticated interaction landscape among the proteins. The resulting PPI network was subsequently analyzed using Cytoscape version 3.10.3, with up-regulated genes represented in orange and down-regulated genes in blue (**Figure 5A**). Hub genes were identified from the PPI network using the cytoHubba plugin in Cytoscape. We applied three algorithms: Maximum Neighborhood Component (MNC), Degree, and Maximal Clique Centrality (MCC). Genes consistently ranked in the top 10 by at least two of these algorithms were selected as candidate hub genes. This comprehensive analysis highlighted the top 10 hub genes with the greatest connectivity, emphasizing their critical roles in maintaining network stability and their potential influence on the pathophysiology of sepsis (**Figure 5B**).

### Identification and exploration of diagnostic biomarkers

**Figure 6:**
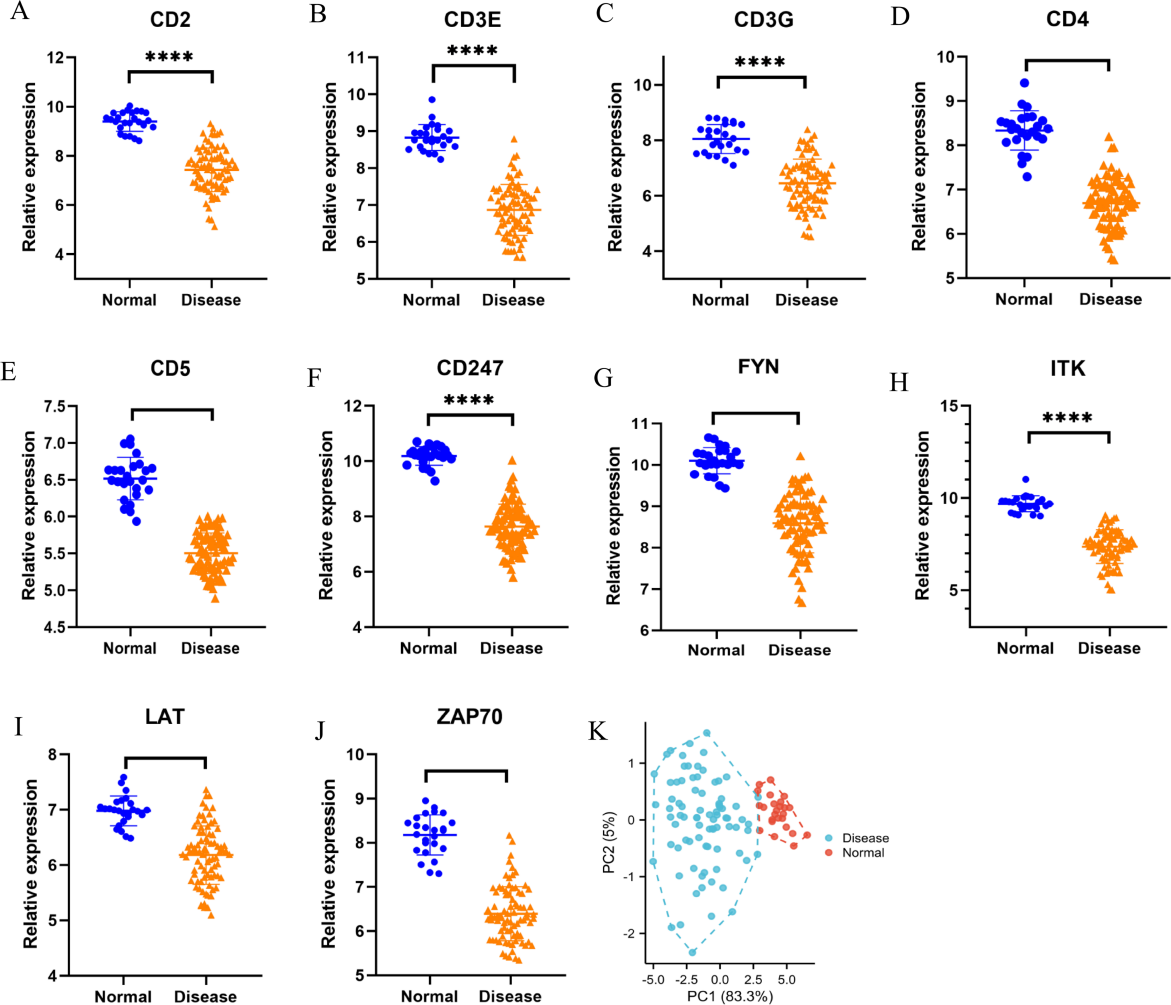
Validation of the identified hub genes. The expression of **(A)** CD2, **(B)** CD3E, **(C)** CD3G, **(D)** CD4, **(E)** CD5, **(F)** CD247, **(G)** FYN, **(H)** ITK, **(I)** LAT, **(J)** ZAP70 and **(K)** PCA between sepsis patients and healthy donors in GSE57065 dataset. *T*-test was employed in the comparison. *P<0.05, **P<0.01, ***P<0.001, ***P< 0.0001.

**Figure 7:**
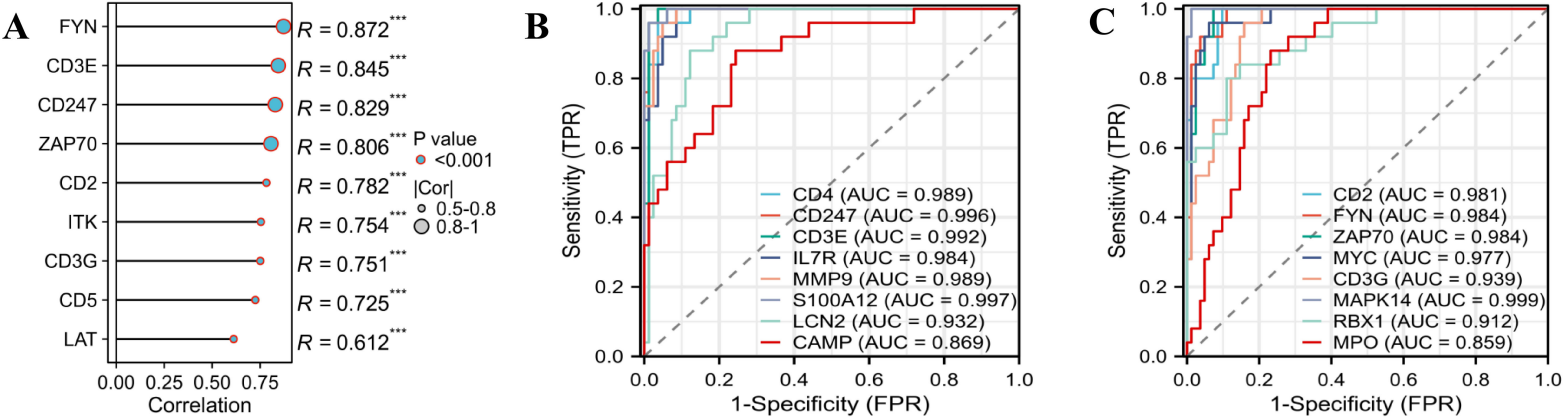
Co-expression correlations and ROC analysis of the ten hub genes in sepsis. **(A)** Co-expression correlation analysis between FYN, CD3E, CD247, ZAP70, CD2, ITK, CD3G, CD5and LAT with CD4. **(B, C)** ROC curves of the ten hub genes inGSE57065. Each biomarker plots one ROC.

To assess the reliability of the ten hub genes, we initially examined their expression within the GSE57065 dataset. As illustrated in **Figures 6A–J**, all identified hub genes exhibited a significant down-regulation in the disease cohort when compared to the normal cohort. PCA depicts the differential expression profiles of sepsis patients versus healthy controls (**Figure 6K**). Furthermore, we conducted a correlation analysis among these genes, which demonstrated strong positive correlations between FYN, CD3E, CD247, and ZAP70 with CD4 (r > 0.8) (**Figure 7A**). To further ascertain the capacity of these genes to discriminate early sepsis, we generated receiver operating characteristic (ROC) curves utilizing the expression data from the GSE57065 dataset. As depicted in **Figures 7B, C**, the area under the curve (AUC) values for CD4, CD247, CD3E, CD2, FYN, ZAP70, CD3G, ITK, LAT, and CD5 were recorded as 0.989, 0.996, 0.992, 0.981, 0.984, 0.984, 0.939, 0.999, 0.908, and 0.998, respectively.

### The relationship between critical genes and immune cell infiltration

**Figure 8:**
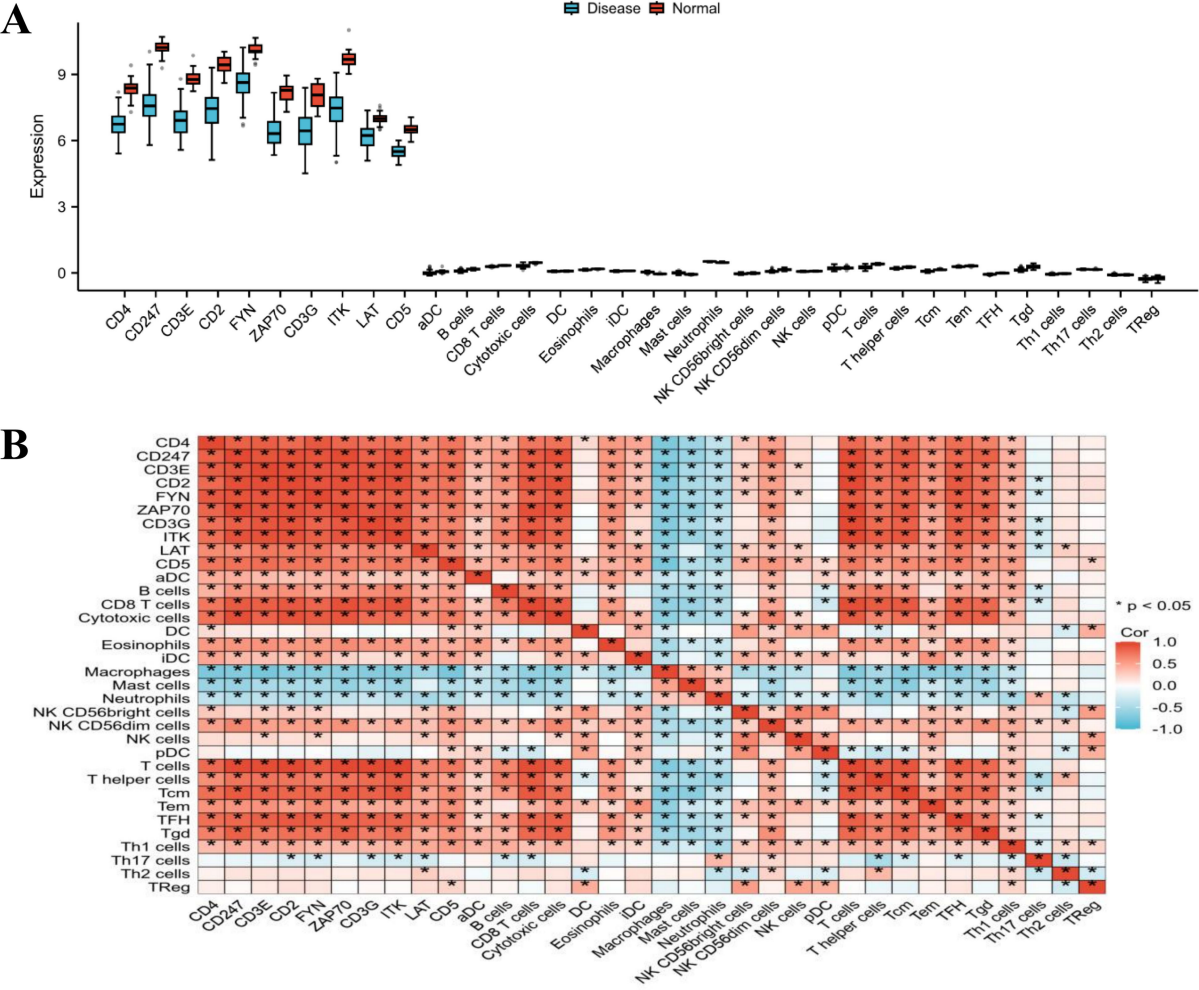
Pertinence of the critical genes with immune cells. **(A)** Boxplots of infiltrating immune cells in GSE57065 dataset. **(B)** Correlations between immune cells and ten critical genes including CD4, CD247, CD3E, CD2, FYN, ZAP70, CD3G, ITK, LAT, and CD5 *P<0.05.

Through the analysis of box plots contrasting the Disease and Normal groups, a significant and coherent downregulation of essential T-cell receptor (TCR) signaling and co-stimulatory molecules—including CD4, CD247, CD3E, CD3G, ZAP70, FYN, ITK, LAT, CD5, and CD2—was observed within the disease context (**Figure 8A**). Notably, all ten genes exhibited reduced median expression levels in the Disease group when compared to the Normal group, aligning with the noted decline in T-cell population. In particular, the fundamental TCR components CD3E, CD3G, and CD247 demonstrated a pronounced reduction, which implies a disruption in either the expression or assembly of the TCR complex. Additionally, the downstream signaling mediators ZAP70, FYN, ITK, and LAT also displayed suppression, indicating a diminished intracellular signaling cascade following antigen interaction. Moreover, the co-stimulatory and adhesion molecules CD2 and CD5, alongside the pivotal T-helper cell marker CD4, were significantly reduced, correlating with the observed decrease in the T-helper cell population depicted in the plot. Taken together, the coordinated down-regulation of these genes offers mechanistic insights into the impaired T-cell presence and functionality noted in the disease samples, underscoring a state of T-cell dysfunction.

The association between the expression of these ten critical T-cell signaling genes and the inferred abundance of various immune cell populations is illustrated through a heatmap. A notable and cohesive pattern emerges from the gene module comprising CD4, CD247, CD3E, CD2, FYN, ZAP70, CD3G, ITK, LAT, and CD5 (**Figure 8B**). These ten genes exhibit strong positive correlations with one another, forming a distinct red cluster, which signifies their coordinated expression within a functional T-cell receptor (TCR) signaling network. Consistently, they display the most robust positive correlations with all T-cell lineages, including general T cells, T helper cells, CD8 T cells, and their respective subsets (Tcm, Tem, Th1, Th17, Th2, and TReg). This observation confirms that elevated expression of these genes constitutes a solid molecular signature indicative of T-cell presence and activity in the microenvironment. Conversely, this gene module reveals neutral to negative correlations with the majority of innate immune and myeloid cells, such as macrophages, neutrophils, activated dendritic cells (aDC), and mast cells. This inverse relationship underscores a potential dichotomy within the immune landscape, where samples enriched in T-cell signals tend to exhibit a proportional decrease in certain myeloid populations, and vice versa. In conclusion, the heatmap substantiates the selected ten genes as a fundamental T-cell-associated signature and elucidates their specific correlation landscape: they serve as synergistic markers for adaptive T-cell immunity while being inversely related to the abundance of several innate immune cell types.

## Discussion

Sepsis and septic shock represent severe medical conditions characterized by a systemic inflammatory response triggered by infections, which can lead to multiple organ failure and elevated mortality rates. According to the World Health Organization, sepsis impacts millions of individuals each year, imposing significant burdens on healthcare systems and resulting in long-term health issues for those who survive (Huynh et al., 2025). The current diagnostic approaches primarily rely on clinical evaluations and non-specific biomarkers (Asif et al., 2025). This often results in delays in timely intervention and effective treatment. The pathophysiology of sepsis is intricate, featuring immune dysregulation that presents as both hyperinflammation and immunosuppression, contributing to considerable variability in patient outcomes (Chen et al., 2023; Liu et al., 2024). Therefore, there is a pressing demand for innovative biomarkers and therapeutic targets that can facilitate early diagnosis and treatment strategies that aim to enhance patient survival and quality of life.

In this investigation, we performed an extensive bioinformatics examination of transcriptomic data obtained from individuals with sepsis to clarify the molecular characteristics associated with immune system dysfunction in this severe clinical state. Through the integration of multiple patient cohorts, we discovered a set of consistently altered genes that are predominantly involved in immune-related biological processes, especially those regulating T-cell activation and functional activity. Analysis of protein-protein interaction networks further identified ten central genes—CD4, CD247, CD3E, CD2, FYN, ZAP70, CD3G, ITK, LAT, and CD5—all of which are recognized elements of the T-cell receptor (TCR) signaling pathway and established identifiers of T-cell lineage (Choi et al., 2024).

The persistent reduction in expression of these central genes across different sepsis cohorts indicates a severe impairment of adaptive immune responses. It is important to emphasize, however, that these genes mainly function as indirect indicators of T-cell quantity rather than direct markers of transcriptional reprogramming specific to sepsis. Their diminished expression is likely a consequence of peripheral T-cell lymphopenia, a widely recognized occurrence in sepsis patients, rather than representing an inherent downregulation of these genes at the individual cell level. This distinction is essential for accurately understanding the biological implications of our results; although the observed expression alterations are consistent and reproducible, they may predominantly reflect changes in immune cell population distribution rather than disease-specific molecular modifications within single T-cells.

This interpretation receives additional support from immune cell infiltration analysis, which showed a strong association between the expression levels of central genes and estimated T-cell abundance. Therefore, the remarkably high diagnostic accuracy demonstrated by these genes (AUC: 0.908–0.999) requires careful consideration. Instead of representing molecular signatures unique to sepsis, these AUC values are probably influenced by variations in immune cell composition between sepsis patients and healthy individuals—specifically, the substantial decrease in circulating T-cells that characterizes sepsis. This does not reduce their clinical value as sensitive indicators of immune system impairment, but it does alter their diagnostic interpretation from disease-specific biomarkers to dependable proxies for immune cell reduction.

The connection between reduced expression of central genes and T-cell exhaustion merits more detailed examination. Although our analysis demonstrated a positive relationship between these genes and markers of exhaustion, this finding may represent two separate phenomena: first, the remaining T-cells in septic patients might display an exhausted state with modified transcriptional profiles; second, the overall decline in T-cell numbers increases the relative presence of exhaustion-related signatures in bulk transcriptomic datasets. Differentiating between these possibilities (cellular depletion versus transcriptional reprogramming) necessitates studies with single-cell resolution that can separate effects due to cell population composition from genuine changes in gene expression within individual cells (Qi et al., 2022).

The Connectivity Map (cMAP) analysis in this study represents an exploratory effort to identify compounds that might reverse the sepsis-associated transcriptomic signature, but several critical limitations should be acknowledged. First, cMAP perturbation data are derived from cancer cell lines (Yu et al., 2023) that fundamentally differ from the primary immune cells driving sepsis pathophysiology. These cell lines lack the complex T-cell receptor signaling machinery central to our hub gene signature, raising concerns about biological relevance to systemic inflammatory diseases. Second, the predicted compounds lack direct mechanistic links to TCR signaling or sepsis-specific immune dysfunction because no evidence connects them to the specific T-cell-related hub genes identified in this study. Third, given our finding that the hub gene signature primarily reflects T-cell abundance rather than intrinsic transcriptional reprogramming, compounds appearing to “reverse” this signature in cancer cell lines may not translate to meaningful restoration of adaptive immunity in septic patients. Despite these limitations, this analysis holds value as a methodological exploration, demonstrating the feasibility of integrating sepsis-associated transcriptomic signatures with computational drug discovery and providing a conceptual framework that highlights the urgent need for immune cell-specific perturbation databases. Substantial experimental validation in primary human T-cells exposed to septic conditions is required before these predictions can inform therapeutic development. In summary, these findings should be interpreted as hypothesis-generating, providing a theoretical reference for future investigations rather than near-term therapeutic candidates for sepsis.

Despite the thorough bioinformatics methodology employed to elucidate sepsis, this study has certain limitations. A significant constraint is the absence of experimental validation of the identified outcomes via experimental laboratory studies, which is vital for corroborating the bioinformatics results. Moreover, owing to the modest sample size, the generalizability of the results should be interpreted with caution. Potential batch effects arising from the amalgamation of multiple Gene Expression Omnibus datasets could also undermine the robustness of the conclusions drawn. Furthermore, the lack of longitudinal data limits our capacity to evaluate temporal changes in gene expression, which may be critical for a more refined comprehension of sepsis progression and its outcomes.

## Conclusion

In summary, this research independently confirms the marked downregulation of T-cell-associated genes during the early stages of sepsis, indicating a severe impairment of adaptive immune function. Utilizing extensive bioinformatics approaches, we detected pivotal differentially expressed genes and pathways, and identified ten central hub genes—all recognized elements of T-cell receptor signaling—that act as reliable indicators of early immune dysregulation. Our main achievement lies not in introducing new diagnostic biomarkers, but in verifying the consistency of these genes as measures of T-cell levels and immune dysfunction in sepsis. These results lay an essential groundwork for upcoming studies focused on T cell–targeted immunomodulatory interventions. Future work should emphasize experimental verification in physiologically appropriate models. Additionally, it should evaluate the practical clinical relevance of these findings to inform immune-directed treatment strategies and enhance patient care in sepsis.

## Data Availability

The original contributions presented in the study are included in the article/**Supplementary Material**. Further inquiries can be directed to the corresponding author.

## References

[B1] Alameer A. Chicco D. (2022). geoCancerPrognosticDatasetsRetriever: a bioinformatics tool to easily identify cancer prognostic datasets on Gene Expression Omnibus (GEO). Bioinf. (Oxford England). 38, 1761–1763. doi: 10.1093/bioinformatics/btab852, PMID: 34935889

[B2] Asif M. Ullah H. Jamil N. Riaz M. Zain M. Pushparaj P. N. . (2025). Advances in diagnostic approaches for alzheimer’s disease: from biomarkers to deep learning technology. CNS neurological Disord. Drug targets. 24, 872–881. doi: 10.2174/0118715273374284250519053646, PMID: 40444626

[B3] Brar S. S. Yathindra M. R. Arango J. S. A. Gutierrez E. A. Aldoohan F. Chahal P. S. . (2025). The narrative review: advancements in heart failure diagnosis and management using artificial intelligence: A new era of patient care. Curr. Cardiol. Rev. doi: 10.2174/011573403x369978250818060357, PMID: 40947703 PMC13519840

[B4] Chen H. Zhang X. Su H. Zeng J. Chan H. Li Q. . (2023). Immune dysregulation and RNA N6-methyladenosine modification in sepsis. Wiley Interdiscip. Rev. RNA. 14, e1764. doi: 10.1002/wrna.1764, PMID: 36149809

[B5] Cheng P. L. Chen H. H. Jiang Y. H. Hsiao T. H. Wang C. Y. Wu C. L. . (2021). Using RNA-seq to investigate immune-metabolism features in immunocompromised patients with sepsis. Front. Med. 8. doi: 10.3389/fmed.2021.747263, PMID: 34977060 PMC8718501

[B6] Choi S. M. Jung K. C. Lee J. I. (2024). Developmental trajectory of unconventional T cells of the cynomolgus macaque thymus. Heliyon. 10, e39736. doi: 10.1016/j.heliyon.2024.e39736, PMID: 39524802 PMC11543906

[B7] Davies K. McLaren J. E. (2024). Destabilisation of T cell-dependent humoral immunity in sepsis. Clin. Sci. (London England: 1979). 138, 65–85. doi: 10.1042/cs20230517, PMID: 38197178 PMC10781648

[B8] Hussain A. Mohammad T. Khan S. Alajmi M. F. Yadav D. K. Hassan M. I. (2025). Seven hub genes associated with huntington’s disease and diagnostic and therapeutic potentials identified by computational biology. Omics: J. Integr. Biol. 29, 154–163. doi: 10.1089/omi.2025.0006, PMID: 40059764

[B9] Huynh C. D. Nguyen P. M. Ngo T. D. Nguyen H. X. Nguyen T. D. Mai H. T. . (2025). Molecular analysis of immune cell subsets and cytokine profiles in septic Vietnamese patients. Clin. Exp. Med. 26, 54. doi: 10.1007/s10238-025-01862-1, PMID: 41348239 PMC12686007

[B10] Kanehisa M. Furumichi M. Sato Y. Matsuura Y. Ishiguro-Watanabe M. (2025). KEGG: biological systems database as a model of the real world. Nucleic Acids Res. 53, D672–D6d7. doi: 10.1093/nar/gkae909, PMID: 39417505 PMC11701520

[B11] Kim K. S. Jekarl D. W. Yoo J. Lee S. Kim M. Kim Y. (2021). Immune gene expression networks in sepsis: A network biology approach. PloS One 16, e0247669. doi: 10.1371/journal.pone.0247669, PMID: 33667236 PMC7935325

[B12] Lin X. M. Zhang L. F. Wang Y. T. Huang T. Lin X. F. Hong X. Y. . (2024). Application of neutrophil-to-lymphocyte-to-monocyte ratio in predicting mortality risk in adult patients with septic shock: A retrospective cohort study conducted at a single center. Heliyon. 10, e28809. doi: 10.1016/j.heliyon.2024.e28809, PMID: 38596065 PMC11002270

[B13] Liu G. Liu Q. Han Z. Wang P. Li Y. (2023). Comparative proteomics analysis of adult Haemonchus contortus isolates from Ovis ammon. Front. Cell. infection Microbiol. 13. doi: 10.3389/fcimb.2023.1087210, PMID: 37009511 PMC10061303

[B14] Liu Z. Ting Y. Li M. Li Y. Tan Y. Long Y. (2024). From immune dysregulation to organ dysfunction: understanding the enigma of Sepsis. Front. Microbiol. 15. doi: 10.3389/fmicb.2024.1415274, PMID: 39252831 PMC11381394

[B15] Lu W. C. Xie H. Yuan C. Li J. J. Li Z. Y. Wu A. H. (2020). Identification of potential biomarkers and candidate small molecule drugs in glioblastoma. Cancer Cell Int. 20, 419. doi: 10.1186/s12935-020-01515-1, PMID: 32874133 PMC7455906

[B16] Muhammad B. A. Hama S. A. Hawrami K. A. M. Karim S. H. Ahmed G. S. Rahim H. M. (2024). Long-term health complications of chemical weapon exposure: a study on Halabja chemical attack survivors (Iraqi Kurds). Inhalation toxicology. 36, 26–30. doi: 10.1080/08958378.2024.2301985, PMID: 38190328

[B17] Qi Y. Han S. Tang L. Liu L. (2022). Imputation method for single-cell RNA-seq data using neural topic model. GigaScience. 12, 1–17. doi: 10.1093/gigascience/giad098, PMID: 38000911 PMC10673642

[B18] Rahmel T. Effinger D. Bracht T. Griep L. Koos B. Sitek B. . (2024). An open-label, randomized controlled trial to assess a ketogenic diet in critically ill patients with sepsis. Sci. Trans. Med. 16, eadn9285. doi: 10.1126/scitranslmed.adn9285, PMID: 38985853

[B19] Reisinger A. C. Posch F. Hackl G. Marsche G. Sourij H. Bourgeois B. . (2021). Branched-chain amino acids can predict mortality in ICU sepsis patients. Nutrients 13, 3106. doi: 10.3390/nu13093106, PMID: 34578983 PMC8469152

[B20] Sahly N. N. Banaganapalli B. Sahly A. N. Aligiraigri A. H. Nasser K. K. Shinawi T. . (2021). Molecular differential analysis of uterine leiomyomas and leiomyosarcomas through weighted gene network and pathway tracing approaches. Syst. Biol. Reprod. Med. 67, 209–220. doi: 10.1093/bioinformatics/btab852, PMID: 33685300

[B21] Tian Y. Wang C. Lu Q. Zhang C. Hu L. Ling J. . (2023). Screening of potential immune-related genes expressed during sepsis using gene sequencing technology. Sci. Rep. 13, 4258. doi: 10.1038/s41598-022-23062-7, PMID: 36918563 PMC10014830

[B22] Wang J. Yin X. Zhang Y. Q. Ji X. (2021). Identification and validation of a novel immune-related four-lncRNA signature for lung adenocarcinoma. Front. Genet. 12. doi: 10.3389/fgene.2021.639254, PMID: 33708243 PMC7940686

[B23] Yang J. Chen C. Jin X. Liu L. Lin J. Kang X. . (2020). Wfs1 and related molecules as key candidate genes in the hippocampus of depression. Front. Genet. 11. doi: 10.3389/fgene.2020.589370, PMID: 33552119 PMC7863986

[B24] Yao R. Q. Li Z. X. Wang L. X. Li Y. X. Zheng L. Y. Dong N. . (2022). Single-cell transcriptome profiling of the immune space-time landscape reveals dendritic cell regulatory program in polymicrobial sepsis. Theranostics. 12, 4606–4628. doi: 10.7150/thno.72760, PMID: 35832091 PMC9254255

[B25] Yu K. Basu A. Yau C. Wolf D. M. Goodarzi H. Bandyopadhyay S. . (2023). Computational drug repositioning for the identification of new agents to sensitize drug-resistant breast tumors across treatments and receptor subtypes. Front. Oncol. 13. doi: 10.3389/fonc.2023.1192208, PMID: 37384294 PMC10294228

