# Supplementary material for: Integrated bioinformatics analysis of molecular signatures and therapeutic targets in early sepsis
Source: Front Cell Infect Microbiol. 2026 Mar 23;16:1802215. doi: 10.3389/fcimb.2026.1802215 (PMC13050901; doi:10.3389/fcimb.2026.1802215)
Supplement: Supplementary file 1 [file Table1.docx]

The Top 10 Hub Genes Analyzed by cytoHubba

| Betweenness | Degree | MCC | MNC |
| --- | --- | --- | --- |
| CD4 | CD4 | CD4 | CD4 |
| FYN | CD247 | CD3E | CD247 |
| ZAP70 | CD2 | CD3G | CD3E |
| CD2 | CD3E | FYN | CD2 |
| ITK | FYN | ITK | CD3G |
| CD247 | CD3G | CD247 | FYN |
| LAT | ZAP70 | ZAP70 | ZAP70 |
| CD3E | CD5 | CD2 | CD5 |
| CD3G | ITK | LAT | ITK |
| CD5 | LAT | CD5 | LAT |
